# The Evolution of the Scavenger Receptor Cysteine-Rich Domain of the Class A Scavenger Receptors

**DOI:** 10.3389/fimmu.2015.00342

**Published:** 2015-07-06

**Authors:** Nicholas V. L. Yap, Fiona J. Whelan, Dawn M. E. Bowdish, G. Brian Golding

**Affiliations:** ^1^Department of Biology, McMaster University, Hamilton, ON, Canada; ^2^Department of Biochemistry and Biomedical Sciences, McMaster University, Hamilton, ON, Canada; ^3^Department of Pathology and Molecular Sciences, McMaster University, Hamilton, ON, Canada

**Keywords:** scavenger receptor, MARCO, SR-A, evolution, selection

## Abstract

The class A scavenger receptor (cA-SR) family is a group of five evolutionarily related innate immune receptors. The cA-SRs are known for their promiscuous ligand binding; as they have been shown to bind bacteria, such as *Streptococcus pneumoniae* and *Escherichia coli*, as well as different modified forms of low-density lipoprotein. Three of the five family members possess a scavenger receptor cysteine-rich (SRCR) domain while the remaining two receptors lack the domain. Previous work has suggested that the macrophage-associated receptor with collagenous structure (MARCO) shares a recent common ancestor with the non-SRCR-containing receptors; however, the origin of the SRCR domain within the cA-SRs remains unknown. We hypothesize that the SRCR domains of the cA-SRs have a common origin that predates teleost fish. Using the newly available sequence data from sea lamprey and ghost shark genome projects, we have shown that MARCO shares a common ancestor with the SRCR-containing proteins. In addition, we explored the evolutionary relationships within the SRCR domain by reconstructing the ancestral SRCR domains of the cA-SRs. We identified a motif that is highly conserved between the cA-SR SRCR domains and the ancestral SRCR domain that consist of WGTVCDD. We also show that the GRAEVYY motif, a functionally important motif within MARCO, is poorly conserved in the other cA-SRs and in the reconstructed ancestral domain. Further, we identified three sites within MARCO’s SRCR domain, which are under positive selection. Two of these sites lie adjacent to the conserved WGTVCDD motif, and may indicate a potential biological function for these sites. Together, these findings indicate a common origin of the SRCR domain within the cA-SRs; however, different selective pressures between the proteins may have caused MARCOs SRCR domain to evolve to contain different functional motifs when compared to the other SRCR-containing cA-SRs.

## 1 Introduction

The scavenger receptors (SRs) are a group of pattern recognition receptors (PRRs), which were originally defined for their ability to bind forms of low-density lipoprotein (LDL) and are subdivided into 8 classes (A–H) (1, 2). These receptors are extracellular glycoproteins, which mediate phagocytosis of negatively charged ligands (3). This binding ability was later refined to include host-modified ligands, such as oxidized LDL (ox-LDL), acetylated LDL (acLDL), and various bacterial ligands, including *Streptococcus pneumoniae*, *Escherichia coli* (4), and *Mycobacterium tuberculosis* (5).

**Figure 1:**
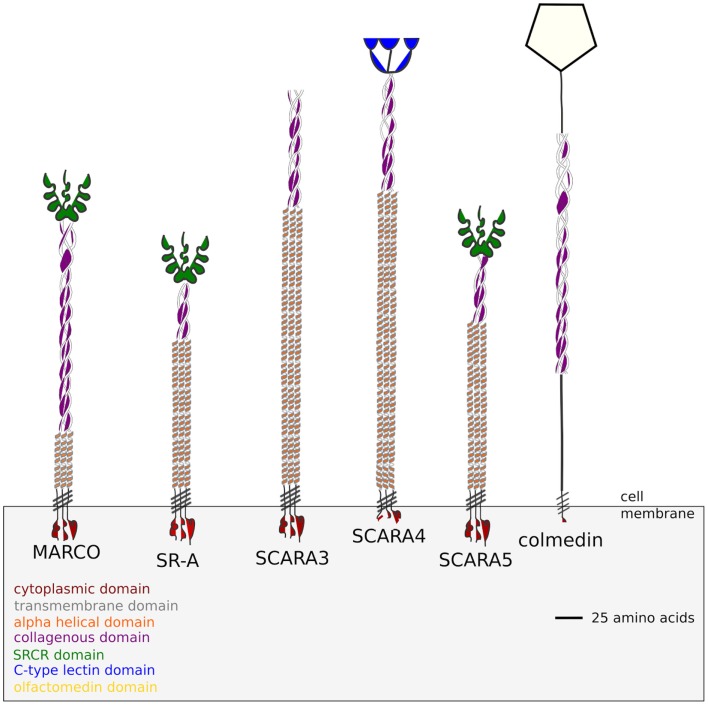
**Domain structure of the five class A Scavenger Receptors based on the protein sequences obtained from the *Homo sapiens* genome**. SCARA3 terminates at its collagenous domain while SCARA4 possesses a C-type Lectin domain. SCARA5, MARCO, and SR-A all possess a terminal SRCR domain. Colmedin is a transmembrane protein with a collagenous and olfactomedin domain found in *Strongylocentrotus purpuratus*, which has been included as an outgroup in this study.

The class A Scavenger Receptors (cA-SRs), one of eight classes of SRs, are membrane-associated phagocytic receptors, which reside on the surface of immune cells (6). The cA-SR family consists of five members: the scavenger receptor class A (SR-A) (4), macrophage-associated receptor with collagenous structure (MARCO) (7), SCAvenger Receptor class A member 3 (SCARA3) or Cellular Stress Response 1 (CSR1) (8), SCAvenger Receptor class A member 4 (SCARA4) or scavenger receptor with C-type lectin domain (SRCL) (9), and SCAvenger Receptor class A member 5 (SCARA5) (10). Despite forming a protein family, the 5 cA-SR proteins differ from each other in a few key ways. First, the 5 receptors are expressed differentially on immune cells. For example, it has been shown in mice, that SR-A is restricted to specific myeloid lineages; however, SCARA5 is expressed exclusively on epithelial cells (10). Further, there are also differences in domain structure between the cA-SRs. MARCO, SR-A, and SCARA5 differ from SCARA3 and SCARA4 in that they possess a scavenger receptor cysteine-rich (SRCR) domain (Figure 1). The SRCR domain is replaced by a C-type lectin domain in SCARA4, while SCARA3 terminates at the collagenous domain. Functionally, MARCO and SR-A both possess SRCR domains, but do not recognize the same ligands. For example, one study identified that the surface proteins of *Neisseria meningitidis* and showed that MARCO and SR-A were able to bind different target proteins (11). MARCO has also been shown to play a functional role in binding *M. tuberculosis*. Polymorphisms within MARCO have been shown to be associated with altered susceptibility to tuberculosis in a Gambian population, whereas no relation was found between infection and polymorphisms in SR-A (5). In addition, MARCO also plays a direct role in host defense during *S. pneumoniae* infection. Using an infection model in mice, MARCO has been shown to be important for cytokine and chemokine production in response to *S. pneumoniae* infection; however, SR-A knock-out mice do not show impaired killing of the bacterium (12). In addition, MARCO is thought to play a role in antigen presentation and/or antigen transfer to dendritic cells and thereby generating T-cell tolerance (13). These suggest an important role for MARCO in host defense, while SR-A is primarily involved in the clearance of modified lipids (5).

Outside of the SRCR domain the five ca-SRs share a similar domain structure, with each protein possessing a cytoplasmic domain, transmembrane domain, and a collagenous domain (Figure 1). All of the cA-SRs possess a cytoplasmic domain, a transmembrane domain, and an alpha helical domain, but they differ in the length of their collagenous domain and at their terminal regions. Despite different ligand binding domains, these receptors share similar ligand binding properties. For instance, all of the receptors except SCARA3 have been shown to bind Gram negative and Gram positive bacteria (1). In addition, SR-A, MARCO, and SCARA4 all bind ox-LDL despite SCARA4 lacking a SRCR domain (1). SR-A and MARCO also share several bacterial binding capabilities including *E. coli* and *Staphylococcus aureus* (3, 7, 14).

The SRCR domain is an evolutionarily conserved 90–110 amino acid long domain that is characterized by 6–8 cysteine residues (3). Within the genome of *Strongylocentrotus purpuratus* (purple sea urchin), over 1200 SRCR domains were identified, often with several domains found in tandem repeats (15). It has been hypothesized that these multiple SRCR domains play a role in cell adhesion, a role shared by some SRCR domains found in vertebrate proteins (16). SRCR domains are classified into two categories: type A domains, which possess six cysteine residues encoded by multiple exons, and type B domains, which contain eight cysteine residues encoded by a single exon (3). These cysteine residues are thought to bind intracellularly, creating three and four disulfide bridges in class A and class B, respectively (17). Three of the cA-SRs possess type A SRCR domains, sharing 6 conserved cysteine residues with other type A SRCR domains. The SRCR domain has been experimentally shown to be required for bacterial binding by utilizing a positively clustered RGR motif within MARCO (18). However, in SR-A, experiments using its isoform, SR-AII, which possesses a truncated SRCR domain, indicate that the SRCR domain is not necessary for the binding of bacterial ligands (19).

Previous work has hypothesized that the cA-SR family members were created through multiple duplication events of an ancestral gene (20). Of the SRCR-containing receptors, SCARA5 and SR-A have been shown to be more closely related to each other than to MARCO. Analyses have shown that the SRCR-containing cA-SRs diverged from SCARA3 and SCARA4, perhaps as early as within the genomes of teleost fish (20). However, the domain structure of the ancestral receptor remains unresolved. Furthermore, MARCO’s relationship to the other cA-SRs is unclear as it contains an SRCR domain that shares functional similarity with SR-A, but appears to share a common ancestor with SCARA3 and SCARA4 (20). With the recently published genomes of *Petromyzon marinus* (sea lamprey) and *Callorhinchus milii* (ghost shark), we can now study the evolution of the cA-SRs before the divergence of teleost fish. In this study, we test the hypothesis that MARCO, SR-A, and SCARA5 share a common ancestor containing the SRCR domain using various phylogenetic approaches. In addition, we reconstruct a hypothetical ancestral SRCR domain and analyze the evolution of two motifs within the SRCR domain. We also test the hypothesis that MARCO’s SRCR domain is under different selective pressure when compared to that of SCARA5 and SR-A due to its direct role in host defense. These data will provide new insight on the origin of the SRCR domain and also its role in ligand binding within the Class A Scavenger Receptors.

## 2 Materials and Methods

### 2.1 Gathering nucleotide and amino acid sequence data

Amino acid sequences of the 5 cA-SRs were searched for using the National Center for Biotechnology Information (NCBI) and the ENSEMBL databases (21). Full-length, partial, and predicted amino acid sequences for all receptors were included in phylogenetic analyses. Amino acid sequences were gathered from a diverse set of species, with as many representatives as possible from fish, birds, and mammals (accessed March 2014). The total number of sequences for each protein was 40 MARCO, 25 SR-A, 40SCARA5, 40 SCARA4, and 40 SCARA3 (Table S1 in Supplementary Material). In addition, PFAM (Protein Families Database) (22) and TMHMM (TransMembrane Hidden Markov Model) (23) were utilized to characterize the domains present within each of the protein sequences. Protein alignments were done using MAFFT (24) due to the numerous collagenous domains of the cA-SRs.

### 2.2 Phylogenetic analysis

In order to study the evolutionary history of the receptors and their SRCR domains, Bayesian phylogenetic analyses were performed using the software MrBayes (25). Several evolutionary models were studied to determine the model best fit to the data set. Each of our data sets was run under a mixed evolutionary model in MrBayes for 1 million generations. PROTTEST (26) was also utilized to search for the model that maximizes the posterior probabilities of the phylogenetic tree, and confirmed the results from MrBayes (Table 1). These results differed for the SRCR-containing proteins; however, due to the inability to implement the Le and Gascuel (LG) model in MrBayes, we ran our analysis using a Whelan and Goldman (WAG) model.

**Table 1:** **Analysis of the best model for each data set of protein alignment based on PROTTEST and running each receptor in MrBayes for 1 million generations under a mixed model**.

Receptors used for analysis	Model predicted from PROTTEST/MrBayes	Support (Log likelihood)
SRCR-containing proteins (MARCO, SR-A, SCARA5)	LG + G/WAG + IG	−6950.09
All 5 cA-SRs (MARCO, SCARA3, SCARA4, SCARA5, SR-A)	JTT + G	−82497.07

*WAG, Whelan and Goldman model with invariable (I) sites and gamma (G) distributed rates; LG + G, Le and Gascuel model with gamma distributed rates; JTT + G, Jones Thorton and Taylor model with gamma distributed rates*.

Analysis of the SRCR domain of SR-A, MARCO, and SCARA5 was carried out using the WAG model with invariable (I) sites and gamma (G) distributed rates for 10 million generations. A combined tree of all 5 cA-SRs was performed using a Jones Thorton and Taylor (JTT) model with an IG distribution for 15 million generations and displayed with midpoint rooting. Finally, an analysis of all 5 cA-SRs with an outgroup was performed in MrBayes using JTT with invariable gamma distribution for 20 million generations. All MrBayes output trees were visualized using TRACER (27) to ensure convergence. Trees were visualized in FigTree (28).

In the reconstruction of a tree for the SRCR domains of MARCO, SCARA5, and SR-A, the ninth (1042–1142) and tenth repeat (1153–1255) of the *Geodia cydonium* (sea sponge) SRCR-containing protein (GCSRCR) (NCBI ID: CAA75175.1) were used as outgroups. The GCSRCR protein’s ninth repeat has been shown to share sequence similarity to both MARCO and SR-A’s SRCR domains (29). We also investigated the tenth repeat of the protein because of a nearby alternative splice site (29). Finally, a phylogenetic tree of all five cA-SRs was constructed with a Colmedin protein sequence from *S. purpuratus* as the outgroup (NCBI ID: NP_001073014.1). Colmedin is a membrane spanning protein containing a transmembrane domain, multiple collagenous domains, and an olfactomedin (30) (Figure 1), which plays a role in the sea urchins innate immune system by aiding in the formation of clots where the skin of the organism has been pierced (31). Due to the similar domain structure to the cA-SRs, Colmedin is a suitable outgroup to the scavenger receptor family.

Predicted ancestral SRCR domain sequences were reconstructed using the FastML webserver (32). The MrBayes generated phylogenetic tree of SCARA5, SR-A, and MARCO was used to reconstruct the ancestral SRCR domains of these proteins and compared.

### 2.3 Motif evolution within the SRCR domain

Consensus sequences of SCARA5 and MARCO were generated for mammals, birds, fish, and other species using Jalview (33) and represented as logos using WebLogo (34). We focused our analysis on two motifs within these proteins; MARCO contains a RGRAEVYY motif (amino acids 440–488 in *Mus musculus*) and a WGTICDD motif (amino acids 452–458 in *M. musculus*) of interest; SCARA5 contains an EGRVEVYH motif (position 399–406) in *M. musculus* and a WGTVCDD motif (position 410–416) in *M. musculus*. We included the RGRAEVYY motif in our analysis due to its known functional role in ligand binding within MARCO (18). WebLogos for MARCO were made using 1 sequence from *P. marinus* (sea lamprey), 1 sequence from *C. milii* (ghost shark), 23 sequences from various mammals, 7 sequences from reptiles, amphibians and birds, and 8 sequences from fish. WebLogos for SCARA5 were made using 1 sequence from *P. marinus* (sealamprey), 26 sequences from various mammals, 6 sequences from reptiles, birds, and amphibians, and 7 fish sequences.

### 2.4 Differences in selective pressure within the SRCR domain

Using the phylogenetic analysis by maximum likelihood (PAML) package, we tested whether MARCO is under a different selective pressure from SCARA5 and SR-A (35). Using codeml (35), we analyzed only the SRCR domain of MARCO to determine any sites under positive selection. We restricted our analysis to include only the SRCR domains from MARCO, SR-A, and SCARA5. To generate our phylogenetic tree, primate sequences were used with *M. musculus* as the outgroup. MARCO, SR-A, and SCARA5 sequences from *M. musculus*, *Homo sapiens*, *Pan troglodytes*, and *Gorilla gorilla* were used. Protein alignments were performed using MAFFT (24) and were subsequently transformed into codon alignments using Pal2nal (36). We used a branch-site model to allow the ratio of non-synononymous substitutions (dN) to synononymous substitutions (dS) to vary along the branches of the tree and the codon sites. We performed a Likelihood Ratio Test (Δ LRT) to determine the significance for the alternative model compared to a null model.

## 3 Results

### 3.1 MARCO shares a more recent common ancestor with the SRCR-containing cA-SRs than with SCARA3 and SCARA4

The evolutionary history of the five cA-SRs has been studied previously (20). In this previous study, it was hypothesized that a single gene duplication event created SCARA5 and SR-A, while MARCO’s relationship to the cA-SRs was left unclear (20). We hypothesize that SCARA3 and SCARA4 were generated through one duplication event, while MARCO may have been generated from a duplication event of a SCARA5/SR-A precursor.

To test this hypothesis, our analysis includes additional protein sequences from divergent taxa and includes sequences from more diverse species. We are able to expand upon this previous work due to recent genome sequencing projects including the sea lamprey and ghost shark. This allowed for our analysis to include in total, 40 MARCO, 25 SR-A, 40 SCARA5, 40 SCARA4, and 40 SCARA3 protein sequences for this analysis. MARCO was found in birds, reptiles, fish, and mammals and a partial sequence was found in *P. marinus* (sea lamprey). The domains of MARCO varied among species, with different numbers of collagen repeats found across different taxa. Within the SRCR domain of MARCO, the RGRAEVYY motif was highly conserved across mammals but less conserved in birds, reptiles, and fish. The SRCR domain of *P. marinus*, however, did not possess the conserved RGRAEVYY motif characteristic of MARCO. SR-A was found in mammals exclusively, except for a sequence found in *Xenopus tropicalis* (western clawed frog). We included it in our analysis and found that the domains of SR-A were highly conserved in both the collagenous and SRCR regions of mammals and the *X. tropicalis* sequence, 70 and 78% conservation, respectively. SCARA5 was found in birds, reptiles, fish, mammals, and as well in *P. marinus*. The collagenous and SRCR domains of SCARA5 were fairly conserved across the 40 species with 69 and 75% conservation, respectively.

**Figure 2:**
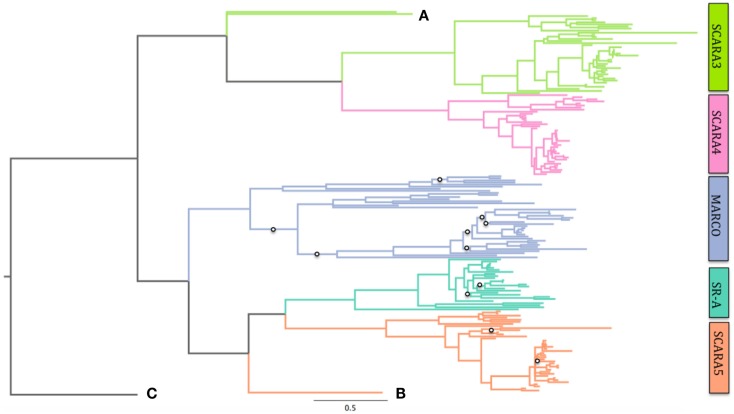
**Phylogeny of all five Class A Scavengers using colmedin as an outgroup**. MARCO branches with SCARA5 and SR-A after rooting on this outgroup. Posterior probabilities for branches with <0.7 confidence are shown with open circles. See Table S1 in Supplementary Material for complete sequence list and accession numbers. Scale bar denotes number of substitutions per site. SCARA3 in sea lamprey (*Petromyzon marinus*) and SCARA3 in southern platyfish (*Xiphophorus maculatus*) are labeled as **(A)** and **(B)** that shows the sea lamprey sequence of SCARA5. These are shown due to their long-branching pattern. **(C)** shows the colmedin sequence.

Using Bayesian phylogenetics, we generated a new phylogenetic tree of the cA-SR family. Here, we show using midpoint rooting that the non-SRCR-containing proteins are more closely related, while the SRCR-containing proteins branch together (Figure S1 in Supplementary Material). Within the SRCR-containing proteins, SCARA5 and SR-A cluster together while MARCO appears to have diverged from them before early teleost fish and possibly before the sea lamprey. We constructed a second phylogenetic tree with the addition of the colmedin sequence as an outgroup. Colmedin was used as it possesses a transmembrane domain and multiple collagenous domains (Figure 2C). Using colmedin as an outgroup, MARCO, SCARA5, and SR-A still cluster together while the non-SRCR-containing receptors form their own branch (Figure 2). Due to the uncertainty regarding the *X. tropicalis* SR-A sequence, we repeated our analysis excluding the sequence and found no difference in our findings (data not shown). These data suggest that MARCO shares a more recent common ancestor with SR-A and SCARA5 than with SCARA3 and SCARA4.

### 3.2 Ancestral reconstruction shows conservation of functional motifs within MARCO and SCARA5, and reveal a common origin for the SRCR domain within the class a scavenger receptors

Our current knowledge of functional motifs within the SRCR domain is limited to the RGRAEVYY motif within MARCO, which contains a positive cluster essential for ligand binding (18). Although we have shown that it is most likely that MARCO, SCARA5, and SR-A share a common origin of the SRCR domain, SCARA5 and SR-A lack the RGRAEVYY motif. We chose to examine whether this motif is specific to MARCO, or if there are similar motifs within the SRCR domains of SR-A and SCARA5. Furthermore, we wanted to determine if there are other conserved motifs between the SRCR domains present in these three receptors. Using FastML (32), we reconstructed the ancestral SRCR domains of MARCO, SR-A, and SCARA5. We focused our analyses on the SRCR domains of MARCO and SCARA5, since SR-A is present primarily in mammals.

**Figure 3:**
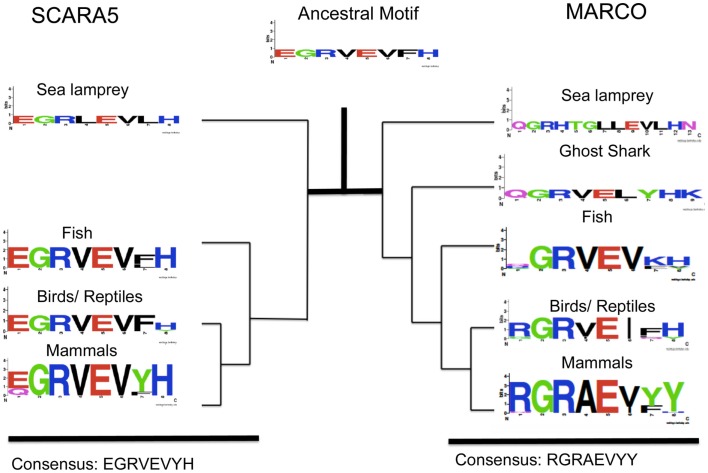
**Evolution of an EGRVEVYH motif within SCARA5 and MARCO’s RGRAEVYY motif among different taxa**. Taxa groups shown include mammals, birds and reptiles, fish, ghost shark, and sea lamprey. The ancestral sequence predicted from FastML is shown as a weblogo. This SCARA5 motif is highly conserved across taxa but MARCO’s motif is less conserved outside of mammals.

Within MARCO’s SRCR domain, amino acids 440–448 (in *M. musculus*) contain the RGRAEVYY motif. The motif is highly conserved within mammals, but is less conserved in fish, where it takes the form of QGRVEVFH (Figure 3). These two motifs are homologous between mammals and fish, but differences in selective pressure may have changed the content of the motif throughout evolution. We reconstructed the ancestral SRCR domain of MARCO that predates the sea lamprey to analyze the domain’s original form. Based on our ancestral reconstruction, the ancestral version of this motif was an EGRVEIFH motif.

We also studied the SRCR domain of SCARA5 across various species to compare with MARCO. Based on our multiple sequence alignment, SCARA5 contains a similar motif to RGRAEVYY. Across all the different species, SCARA5 contains a highly conserved EGRVEVYH motif, where only the first glutamic acid (E), tyrosine (Y), and histidine (H) are somewhat variable (Figure 3). We also constructed an ancestral SCARA5 sequence, which contained a motif of the form EGRVEVFH. Interestingly, the MARCO motif, QGRVEVKH, within fish resembles the EGRVEVFH motif of SCARA5. These suggest that the RGRAEVYY motif is specific to mammalian MARCO proteins, and may be under selective pressure due to its role in bacterial binding. In addition, the ancestral motif to both SCARA5 and MARCO most likely took the form of an EGRVEVFH motif (Figure 3).

**Figure 4:**
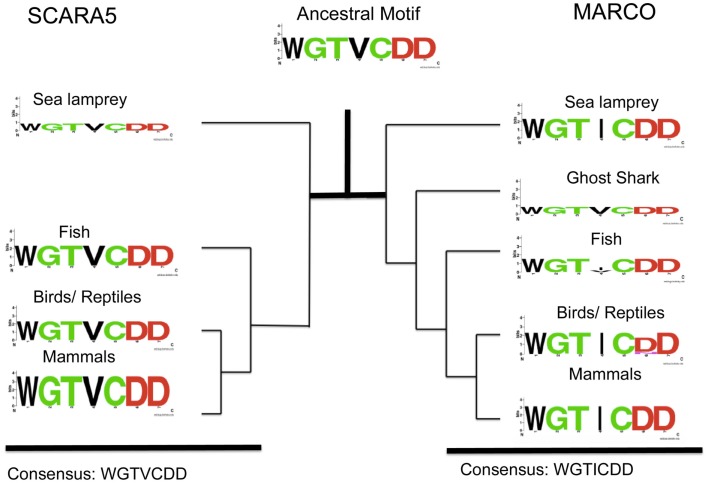
**Analysis of SCARA5’s WGTVCDD motif and MARCO’s WGTICDD motif within different taxa**. Taxa groups shown include mammals, birds and reptiles, fish, ghost shark, and sea lamprey. The ancestral sequence predicted from FastML is shown. The WGTVCDD motif is conserved between the ancestral proteins and within both SCARA5 and MARCO except at the valine residue (site 4 in the motif).

We also identified another highly conserved motif consisting of WGTICDD in MARCO at amino acids 452–458 in *M. musculus*. This newly identified motif is highly conserved across the SRCR domains of MARCO, SR-A, and SCARA5, and we hypothesize that the motif may have a functional role due its proximity to a conserved cysteine residue (C1) within the SRCR domain. In contrast to the RGRAEVYY motif, the WGTICDD motif within MARCO is highly conserved across all the different taxa examined (Figure 4). The isoleucine residue, position 445 of MARCO in *M. musculus*, is the only variable site, which is replaced by a valine (V) in some fish species. Based on our multiple sequence alignment, SCARA5 possesses a homologous, highly conserved, WGTVCDD (Figure 4). Interestingly, within fish, the sea lamprey, and the ghost shark, MARCO’s WGTICDD motif resembles the WGTVCDD motif of SCARA5. Taken together with our previous finding, both the RGRAEVYY motif and the WGTICDD motif within MARCO resembles those within SCARA5; however, the two motifs may be under different selective pressures. These suggest that the two motifs were found in the ancestral SRCR domain and most likely resembled an EGRVEVFH and WGTVCDD motif, respectively.

### 3.3 Evidence of positive selection within the SRCR domain of MARCO

MARCO’s SRCR has been shown to play a direct role in ligand binding and host immunity defense (5). Previous work has shown the importance of arginine residues (R) within the SRCR domain (18), as well as the RGRAEVYY motif (7). Due to MARCO’s direct role in binding various bacteria including *S. pneumoniae* (37) and *E. coli* (14), and an uncertain role in SCARA5, we hypothesize that MARCO’s SRCR domain is under positive selection.

**Figure 5:**
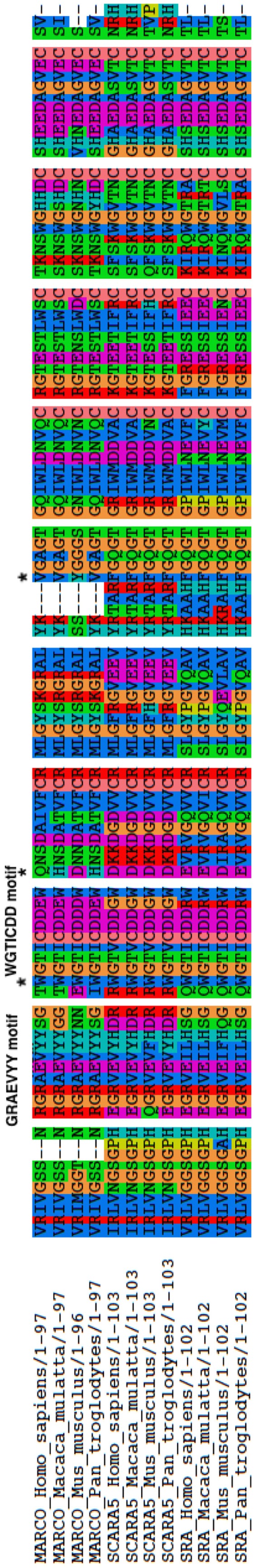
**Alignment of the SRCR domains studied for positive selection test in PAML**. The GRAEVYY motif and WGTICDD motif, found in MARCO, are labeled. Stars denote positions 442, 452, and 477 as sites identified as under positive selection from PAML.

We tested for positive selection within MARCO using a branch-site model, where the ratio of non-synonymous substitutions (dN) to synonymous substitutions (dS) is free to vary along both the branches and the sites of the phylogeny. Using this model, we found evidence for positive selection along several sites within MARCO’s SRCR domain. We confirmed our results with a Likelihood Ratio Test (Δ LRT) at a 95% significance level. The sites identified as under positive selection were 442, 452, and 477 (Table 2). In humans, these sites correspond to tryptophan, glutamine, and valine, respectively. Sites 442 and 452 are of particular interest because of their close proximity to the RGRAEVYY and WGTICDD motifs of MARCO (431–438, 442–448 in mouse, respectively) (Figure 5). Tryptophan 442 corresponds to the first residue of the WGTICDD motif, and could be an indication of positive selection acting on this motif. Due to the high conservation of this motif across all of the SRCR domains, this suggests a potential biological function for T442 and Q452. We also identified position 477 as possibly being under positive selection. Although position 477 is not within close proximity to any of the known motifs, the site had a relatively high BEB score and may have some uncharacterized biological function.

**Table 2:** **Identified sites putatively under positive selection within MARCO’s SRCR domain from PAML**.

Site identified with respect to full-length human MARCO	Bayes empirical Bayes (BEB) score
W 442	0.700
Q 452	0.976
V 477	0.861

## 4 Discussion

The Class A Scavenger Receptor family is a diverse group of Pattern Recognition Receptors involved in innate immunity. Previous work has suggested that the five family members were generated through multiple duplication events; however, several questions remained unanswered. It was unclear whether MARCO shared a more recent common ancestor with the SRCR-containing receptors (SCARA5 and SR-A), or with the non-SRCR-containing receptors (SCARA3 and SCARA4) (20). In addition, the ancestral domains were also unresolved because of the uncertainty regarding MARCO’s relationship to the other cA-SRs (20). Here, we present new phylogenetic data to resolve these uncertainties within the scavenger receptor family.

Using Bayesian methods, we generated a new phylogenetic tree of all five cA-SRs. Our phylogenetic tree shows that MARCO shares a more recent common ancestor with SCARA5 and SR-A than with SCARA3 and SCARA4. The addition of an outgroup sequence, colmedin from *S. purpuratus*, also shows the same relationship. This suggests that an ancestral cA-SR containing an SRCR domain was duplicated to produce an ancestral MARCO and a SCARA5/SR-A like precursor. Following this duplication event, the SCARA5/SR-A like precursor underwent a duplication event to produce modern SCARA5 and SR-A sequences. Based on the available sequence data, it still remains unknown if the ancestral gene to all five cA-SRs lacked or contained the SRCR domain. It is possible that the ancestral gene terminated at its collagenous domain and resembled SCARA3 and later acquired the SRCR domain in the MARCO/SCARA5/SR-A precursor. Equally likely, the ancestral gene may have contained the SRCR domain and it was lost in a SCARA3/SCARA4 precursor. Additional sequence data are required to fully uncover the origin of these proteins.

To investigate the origin of the SRCR domains, we utilized Bayesian methods to construct a phylogenetic tree and subsequently reconstructed ancestral SRCR domains. Based on our analysis, MARCO shares a recent common ancestor with SCARA5 and SR-A. Using FastML, we reconstructed the SRCR domains at ancestral nodes between SCARA5, MARCO, and SR-A. We focused on two motifs within SCARA5 and MARCO; the RGRAEVYY motif within MARCO and a downstream WGTICDD motif. SCARA5 possesses two motifs similar to these, as it contains an EGRVEVYH motif and a WGTVCDD motif. Based on our multiple sequence alignment, the RGRAEVYY motif is specific to MARCO while the WGTVCDD motif is shared between the three SRCR domains. Furthermore, the ancestral motif to all three cA-SR SRCR domains resembled SCARA5’s EGRVEVYH and WGTVCDD motifs. This suggests that MARCO’s SRCR domain originally resembled SCARA5, and may have undergone purifying selection. Since SCARA5 and the ancestral domain lack the RGRAEVYY motif, we hypothesize the ancestral SRCR domain did not play a functional role in ligand binding due to the functional importance of the RGRAEVYY motif within MARCO. In invertebrate species, the SRCR domains are thought to play a role in cellular recognition as opposed to ligand binding (16). This theory may also apply to the original SRCR domain of the cA-SRs.

Given MARCO’s role in bacterial binding and clearance within the immune system, we hypothesized that its SRCR domain may be under positive selection. Here, we have shown that MARCO has several sites under positive selection including positions 442, 452, and 477. Two of the sites are adjacent to the highly conserved WGTVCDD motif within the SRCR domain, and may have a biological function. However, we did not detect positive selection acting on the RGRAEVYY motif, despite the domain having a known role in ligand binding. Future experiments will look to identify the functional relevance of the positively selected sites and the WGTVCDD motif. We hypothesize that the WGTVCDD motif may have a structural role within the SRCR domain due to the inclusion of a conserved cysteine within the motif. Studying WGTICDD motif will further our understanding of the SRCR domain and its biological relevance in MARCO’s ability to facilitate bacterial binding, phagocytosis, and induction of T-cell tolerance.

## Author Contributions

Manuscript planned and written by all. NY conducted and implemented all experiments.

## Conflict of Interest Statement

The authors declare that the research was conducted in the absence of any commercial or financial relationships that could be construed as a potential conflict of interest.

## Supplementary Material

The Supplementary Material for this article can be found online at http://journal.frontiersin.org/article/10.3389/fimmu.2015.00342
