# Supplementary material for: The Evolution of the Scavenger Receptor Cysteine-Rich Domain of the Class A Scavenger Receptors
Source: Front Immunol. 2015 Jul 6;6:342. doi: 10.3389/fimmu.2015.00342 (PMC4491621; doi:10.3389/fimmu.2015.00342)
Supplement: Supplementary file 1 [file Table_1.PDF]

Supplementary Table 1: Accession Numbers of Sequences of the cA-SRs used for Bayesian analysis and PAML

Accession Numbers	*Organism*	cA-SR
XP_004481938	*Dasypus novemcinctus*	SR-A
XP_002756952	*Callithrix jacchus*	SR-A
XP_848261	*Canis lupus*	SR-A
XP_001488613	*Equus caballus*	SR-A
XP_002928655	*Ailuropoda melanoleuca*	SR-A
EHB08261	*Heterocephalus glaber*	SR-A
XP_003412472	*Loxodonta africana*	SR-A
XP_001097884	*Macaca mulatta*	SR-A
XP_001374095	*Monodelphis domestica*	SR-A
NP_619729	*Homo sapiens*	SR-A
NP_001106797	*Mus musculus*	SR-A
ELK37699	*Myotis davidii*	SR-A
XP_003256747	*Nomascus leucogenys*	SR-A
XP_004277216	*Orcinus orca*	SR-A
XP_001512876	*Ornithorhynchus anatinus*	SR-A
NP_001075717	*Oryctolagus cuniculus*	SR-A
XP_003797724	*Otolemur garnettii*	SR-A
XP_004021785	*Ovis aries*	SR-A
XP_001140701	*Pan troglodytes*	SR-A
NP_001230803	*Sus scrofa*	SR-A
NP_001178868	*Rattus norvegicus*	SR-A
XP_003772876	*Sarcophilus harrisii*	SR-A
NP_001106711	*Bos taurus*	SR-A
XP_004324021	*Tursiops truncatus*	SR-A
ENSXETP00000037776	*Xenopus Tropicalis*	SR-A
XP_004469503	*Dasypus novemcinctus*	MARCO
AFK11537	*Callorhinchus milii*	MARCO
XP_003478655	*Cavia porcellus*	MARCO
NP_990067	*Gallus gallus*	MARCO
EMC87396	*Columba livia*	MARCO
EGV99069	*Cricetulus griseus*	MARCO
XP_533324	*Canis lupus*	MARCO
XP_002917620	*Ailuropoda melanoleuca*	MARCO
XP_003416719	*Loxodonta africana*	MARCO
XP_001083118	*Macaca mulatta*	MARCO
NP_006761	*Homo sapiens*	MARCO
XP_002749572	*Callithrix jacchus*	MARCO

XP_004572010	*Maylandia zebra*	MARCO
XP_003207755	*Meleagris gallopavo*	MARCO
Q9WUB9	*Mesocricetus auratus*	MARCO
XP_001368478	*Monodelphis domestica*	MARCO
NP_034896	*Mus musculus*	MARCO
ELK25702	*Myotis davidii*	MARCO
XP_003456263	*Oreochromis niloticus*	MARCO
XP_003275234	*Nomascus leucogenys*	MARCO
XP_004276557	*Orcinus orca*	MARCO
XP_002712449	*Oryctolagus cuniculus*	MARCO
XP_004086950	*Oryzias latipes*	MARCO
XP_515756	*Pan troglodytes*	MARCO
NP_001243295	*Sus scrofa*	MARCO
XP_002812467	*Pongo abelii*	MARCO
NP_001102481	*Rattus norvegicus*	MARCO
AGH27725	*Sciaenops ocellatus*	MARCO
XP_004004798	*Ovis aries*	MARCO
XP_869408	*Bos taurus*	MARCO
XP_002944607	*Xenopus tropicalis*	MARCO
XP_004175222	*Taeniopygia guttata*	MARCO
ENSLACP00000019789	*Latimeria chalumnae*	MARCO
ENSGACP00000001962	*Gasterosteus aculeatus*	MARCO
ENSDARP00000076803	*Danio rerio*	MARCO
ENSPSIP00000003188	*Pelodiscus sinensis*	MARCO
ENSPMAP00000008064	*Petromyzon marinus*	MARCO
GAFZ01108062	*Anolis Carolinensis*	MARCO
GAIB01020145_1	*Nothobranchius furzeri*	MARCO
XP_004315380	*Tursiops truncatus*	MARCO
EOA93253	*Anas platyrhynchos*	SCARA3
XP_004454217	*Dasypus novemcinctus*	SCARA3
XP_614788	*Bos taurus*	SCARA3
XP_002807525	*Callithrix jacchus*	SCARA3
XP_543225	*Canis lupus*	SCARA3
EMP24884	*Chelonia mydas*	SCARA3
XP_693010	*Danio rerio*	SCARA3
XP_001492909	*Equus caballus*	SCARA3
XP_001234416	*Gallus gallus*	SCARA3
NP_057324	*Homo sapiens*	SCARA3
XP_001110670	*Macaca mulatta*	SCARA3
XP_004550616	*Maylandia zebra*	SCARA3
XP_003204626	*Meleagris gallopavo*	SCARA3

XP_001380024	*Monodelphis domestica*	SCARA3
NP_766192	*Mus musculus*	SCARA3
ELK29387	*Myotis davidii*	SCARA3
XP_003446373	*Oreochromis niloticus*	SCARA3
XP_003272990	*Nomascus leucogenys*	SCARA3
XP_004270781	*Orcinus orca*	SCARA3
XP_002709349	*Oryctolagus cuniculus*	SCARA3
XP_001515573	*Ornithorhynchus anatinus*	SCARA3
XP_004084013	*Oryzias latipes*	SCARA3
XP_003794065	*Otolemur garnettii*	SCARA3
XP_519678	*Pan troglodytes*	SCARA3
XP_002818982	*Pongo abelii*	SCARA3
NP_001102340	*Rattus norvegicus*	SCARA3
XP_003971950	*Takifugu rubripes*	SCARA3
XP_003757833	*Sarcophilus harrisii*	SCARA3
XP_003359089	*Sus scrofa*	SCARA3
ELW62541	*Tupaia chinensis*	SCARA3
XP_002938223	*Xenopus tropicalis*	SCARA3
ENSACAT00000015059	*Anolis carolinensis*	SCARA3
ENSPSIT00000005080	*Pelodiscus sinensis*	SCARA3
ENSGMOT00000004583	*Gadus morhua*	SCARA3
ENSLACT00000011225	*Latimeria chalumnae*	SCARA3
ENSPMAT00000007605	*Petromyzon marinus*	SCARA3
ENSXMAT00000000548	*Xiphophorus maculatus*	SCARA3
ENSGACT00000010151	*Gasterosteus aculeatus*	SCARA3
ENSTRUT00000033803	*Takifugu rubripes*	SCARA3
ENSTNIT00000015378	*Tetraodon nigroviridis*	SCARA3
EOB08780	*Anas platyrhynchos*	SCARA4
XP_003219726	*Anolis carolinensis*	SCARA4
XP_004447445	*Dasypus novemcinctus*	SCARA4
NP_001095313	*Bos taurus*	SCARA4
XP_002757150	*Callithrix jacchus*	SCARA4
XP_849057	*Canis lupus*	SCARA4
XP_003474092	*Cavia porcellus*	SCARA4
XP_003507186	*Cricetulus griseus*	SCARA4
NP_001116312	*Danio rerio*	SCARA4
CBN82070	*Dicentrarchus labrax*	SCARA4
XP_001492967	*Equus caballus*	SCARA4
NP_001034688	*Gallus gallus*	SCARA4
XP_002922671	*Ailuropoda melanoleuca*	SCARA4
NP_569057	*Homo sapiens*	SCARA4

XP_003406821	*Loxodonta africana*	SCARA4
XP_001088438	*Macaca mulatta*	SCARA4
XP_004546924	*Maylandia zebra*	SCARA4
XP_003205037	*Meleagris gallopavo*	SCARA4
XP_001368023	*Monodelphis domestica*	SCARA4
NP_569716	*Mus musculus*	SCARA4
ELK31640	*Myotis davidii*	SCARA4
XP_003439401	*Oreochromis niloticus*	SCARA4
XP_003262063	*Nomascus leucogenys*	SCARA4
ABV44703	*Oncorhynchus mykiss*	SCARA4
XP_004273818	*Orcinus orca*	SCARA4
XP_001508422	*Ornithorhynchus anatinus*	SCARA4
XP_002713638	*Oryctolagus cuniculus*	SCARA4
XP_004081176	*Oryzias latipes*	SCARA4
XP_003784843	*Otolemur garnettii*	SCARA4
XP_524004	*Pan troglodytes*	SCARA4
XP_002828148	*Pongo abelii*	SCARA4
NP_001020892	*Rattus norvegicus*	SCARA4
XP_003968105	*Takifugu rubripes*	SCARA4
XP_002194661	*Taeniopygia guttata*	SCARA4
ELW63801	*Tupaia chinensis*	SCARA4
XP_004319786	*Tursiops truncatus*	SCARA4
XP_004411262	*Odobenus rosmarus*	SCARA4
XP_002934169	*Xenopus tropicalis*	SCARA4
GSTENT10018049001	*Tetraodon nigroviridis*	SCARA4
EOA94326	*Anas platyrhynchos*	SCARA5
XP_002756852	*Callithrix jacchus*	SCARA5
XP_003479729	*Cavia porcellus*	SCARA5
XP_001234366	*Gallus gallus*	SCARA5
EGW14787	*Cricetulus griseus*	SCARA5
XP_543223	*Canis lupus*	SCARA5
XP_002914457	*Ailuropoda melanoleuca*	SCARA5
EHB14244	*Heterocephalus glaber*	SCARA5
NP_776194	*Homo sapiens*	SCARA5
XP_003412507	*Loxodonta africana*	SCARA5
XP_002805343	*Macaca mulatta*	SCARA5
XP_004550682	*Maylandia zebra*	SCARA5
XP_003204623	*Meleagris gallopavo*	SCARA5
XP_001370534	*Monodelphis domestica*	SCARA5
NP_083179	*Mus musculus*	SCARA5
ELK29382	*Myotis davidii*	SCARA5

XP_003456669	*Oreochromis niloticus*	SCARA5
XP_003272963	*Nomascus leucogenys*	SCARA5
XP_004270748	*Orcinus orca*	SCARA5
XP_001507091	*Ornithorhynchus anatinus*	SCARA5
XP_002709496	*Oryctolagus cuniculus*	SCARA5
XP_003794062	*Otolemur garnettii*	SCARA5
XP_519680	*Pan troglodytes*	SCARA5
XP_003132867	*Sus scrofa*	SCARA5
XP_002818988	*Pongo abelii*	SCARA5
NP_001129327	*Rattus norvegicus*	SCARA5
XP_004433598	*Ceratotherium simum*	SCARA5
XP_003757828	*Sarcophilus harrisii*	SCARA5
XP_004004485	*Ovis aries*	SCARA5
NP_001095969	*Bos taurus*	SCARA5
CAG12980	*Tetraodon nigroviridis*	SCARA5
XP_004382241	*Trichechus manatus*	SCARA5
EMP24889	*Chelonia mydas*	SCARA5
XP_002941495	*Xenopus tropicalis*	SCARA5
NP_001025361	*Danio rerio*	SCARA5
ENSACAT00000014115	*Anolis carolinensis*	SCARA5
ENSGMOT00000002420	*Gadus morhua*	SCARA5
ENSPMAT00000006907	*Petromyzon marinus*	SCARA5
ENSXMAT00000006370	*Xiphophorus maculatus*	SCARA5
ENSGACT00000009202	*Gasterosteus aculeatus*	SCARA5
