# Supplementary material for: The Evolution of the Scavenger Receptor Cysteine-Rich Domain of the Class A Scavenger Receptors
Source: Front Immunol. 2015 Jul 6;6:342. doi: 10.3389/fimmu.2015.00342 (PMC4491621; doi:10.3389/fimmu.2015.00342)
Supplement: Supplementary file 2 [file Image_1.PDF]

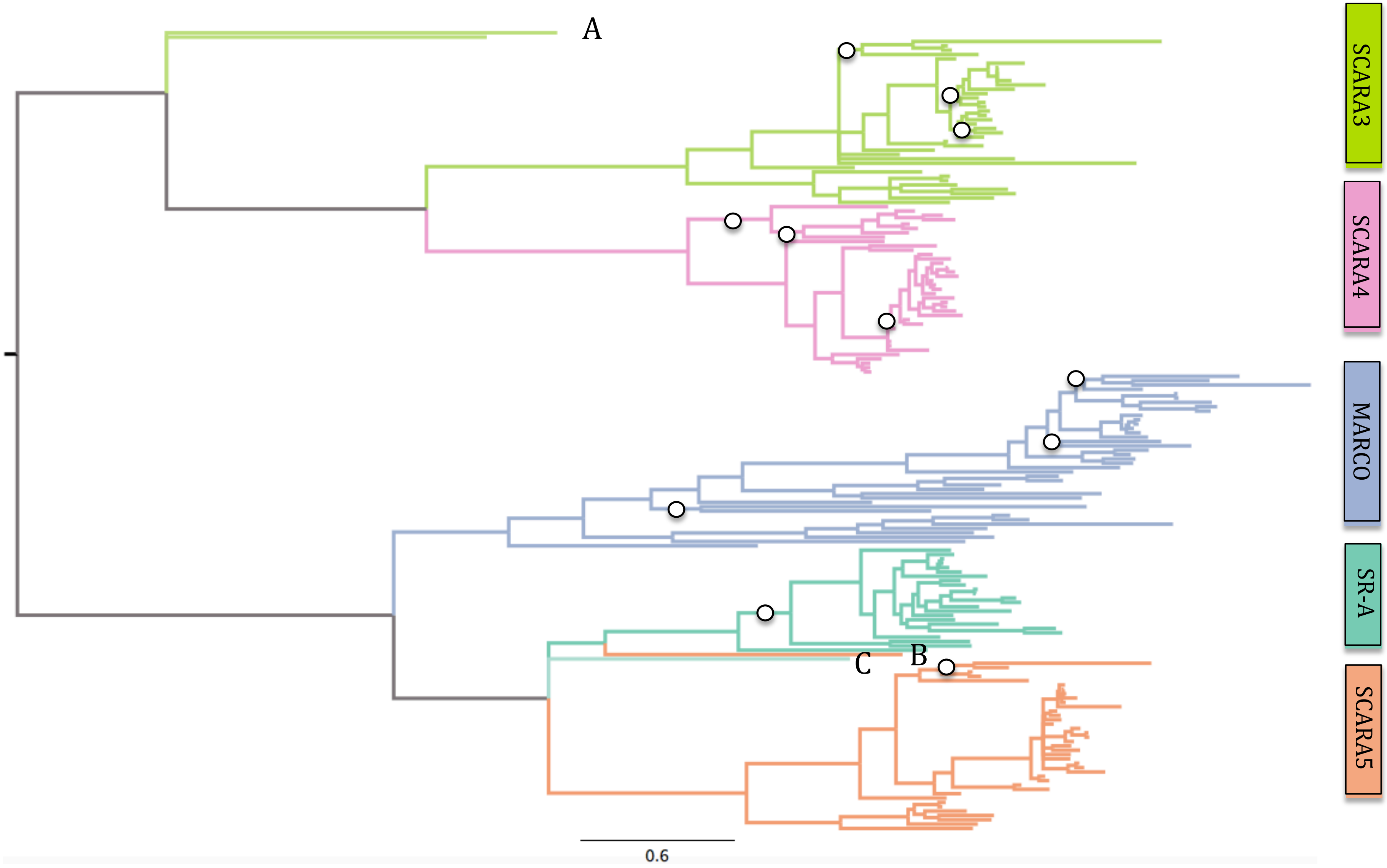


**Supplementary Figure S1**: MrBayes phylogeny of all five Class A Scavenger Receptors using midpoint root. MARCO branches with SCARA5 and SR-A which suggests a common ancestor between the three proteins. Posterior probabilities less than 0.7 are shown with open circles on their respective branches. Scale bar denotes number of substitutions per site. SCARA3 sequences in sea lamprey (*Petromyzon marinus*) and southern platyfish (*Xiphophorus maculatus*) are denoted as A. The sea lamprey SCARA5 sequence is shown by label B and the western clawed frog (*Xenopus tropicalis*) SR-A sequence is labeled as C. These are shown due to their long branching pattern.
