# Supplementary material for: The Evolution of the Scavenger Receptor Cysteine-Rich Domain of the Class A Scavenger Receptors
Source: Front Immunol. 2015 Jul 6;6:342. doi: 10.3389/fimmu.2015.00342 (PMC4491621; doi:10.3389/fimmu.2015.00342)
Supplement: Supplementary file 3 [file Image_2.PDF]

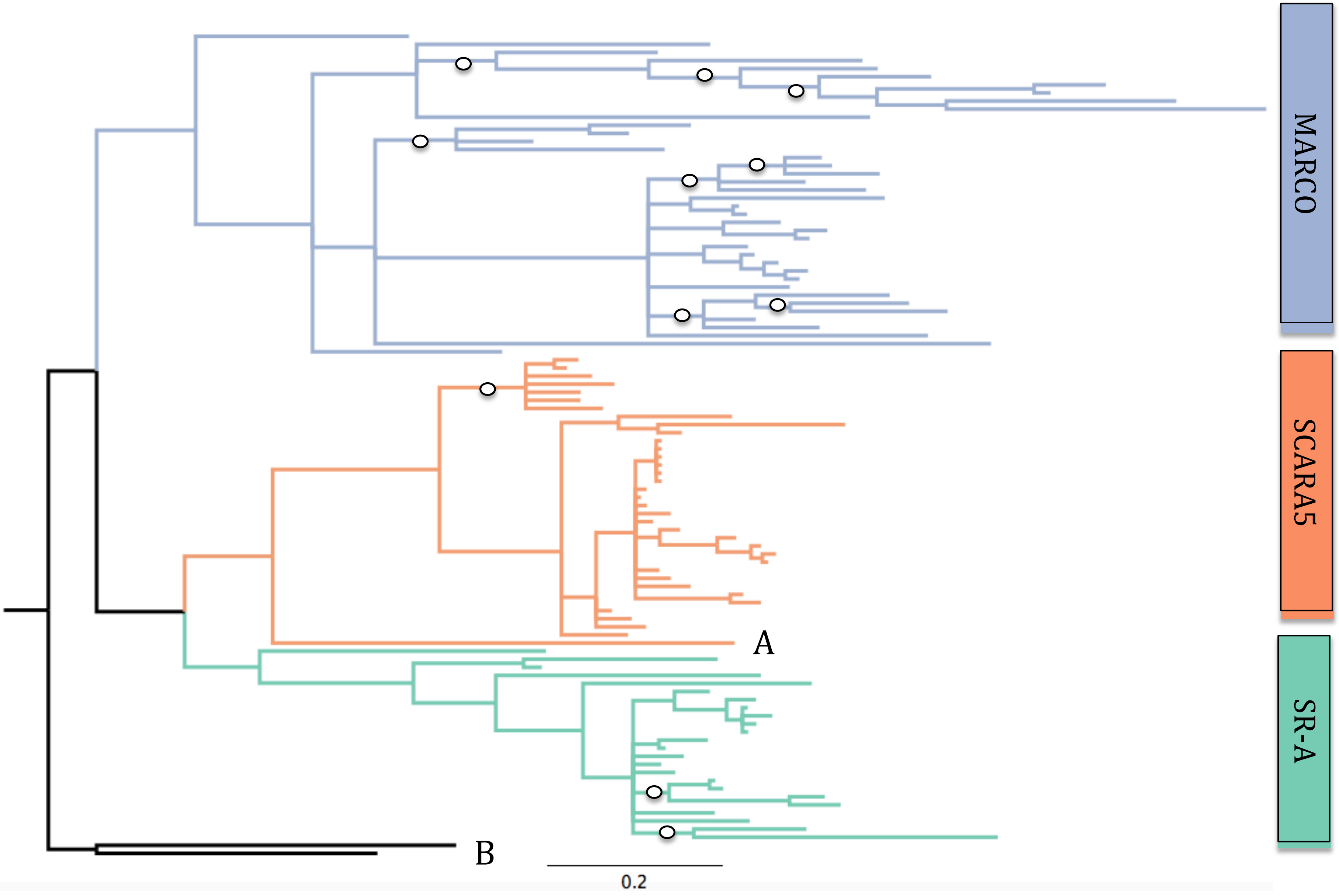

Supplementary Figure S2: The evolutionary relationship between the SRCR-containing Class A Scavenger Receptors using the GCSRCR ninth and tenth SRCR domain repeats as outgroups (label B). The GCSRCR is an SRCR-containing protein found in *Geodia cydonium* (sea sponge). Phylogenetic analysis was performed in MrBayes, with posterior probabilities less than 0.7 are labeled with open circles on their respective branches. Scale bar denotes number of substitutions per site. Label A shows the SCARA5 sequence of sea lamprey (*Petromyzon marinus*).
